# Gene regulatory complexes: their role and regulation across normal and malignant hematopoiesis

**DOI:** 10.1016/j.exphem.2025.104821

**Published:** 2025-06-14

**Authors:** Gina Sangha, Brian J.P. Huntly

**Affiliations:** a Department of Haematology, University of Cambridge, Cambridge, Cambs, United Kingdom; b Cambridge Stem Cell Institute, Cambridge, Cambs, United Kingdom; c Department of Haematology, Cambridge University Hospitals, Cambridge, Cambs, United Kingdom

## Abstract

Transcription is regulated in a multitude of ways to ensure lineage- and context-specific gene expression in a coordinated fashion. Hematopoiesis is an exemplary process for studying the mechanisms of tightly regulated activation and repression of gene expression programs through transcription and gene regulatory complexes. These complexes act by posttranslational modification of histones and nonhistone proteins, epigenetic modifications of DNA, ATP-dependent chromatin remodeling, scaffolding and recruitment of combinatorial protein complexes, and alteration of three-dimensional genome conformation to bring about lineage-specific gene expression. This review will focus on the function of these gene regulatory complexes in hematopoiesis and how they are hijacked in acute myeloid leukemia, highlighting therapeutic progress and opportunities.

**Figure:**
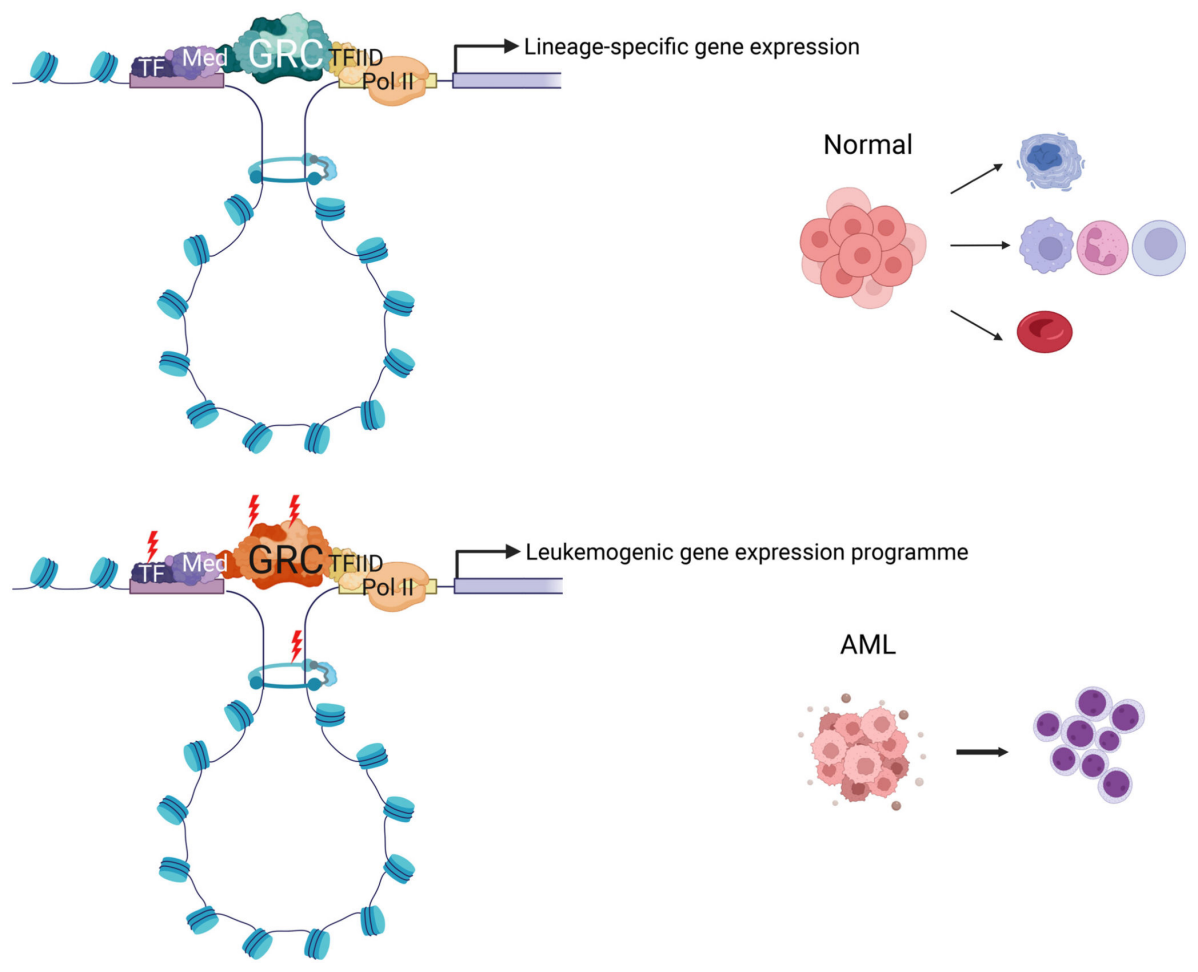

## Chromatin Architecture within Normal Hematopoiesis

Hematopoietic stem and progenitor cells (HSPCs) are defined by their capacities for self-renewal and multipotency, allowing for the full reconstitution of the entire blood system and its maintenance over the lifespan of the organism [1]. Lineage commitment during hematopoiesis requires tightly regulated activation and repression of gene expression programs by individual and combinatorial transcription factors (TFs) in a spatial and temporal-specific manner [2−5]. However, recent evidence points to a complex interplay between TFs and chromatin-regulatory cofactors (CFs) that act by posttranslational histone modification or epigenetic DNA modification, or via ATP-dependent alteration of chromatin accessibility to alter the chromatin landscape in a dynamic and context-specific way. Transcription is further regulated by three-dimensional (3D) genome conformation mediated via genome structural proteins and chromatin modifications that allow cis-regulatory elements, such as enhancers, to influence the activity of promoters in a proximity-mediated manner [6−8]. Maps of DNA methylation, histone modification, and chromatin accessibility have been generated for subpopulations within the hematopoietic hierarchy, revealing changes in chromatin configuration upon HSPC lineage commitment resulting in lineage-specific gene expression [9−12]. Further evidence of the importance of tightly regulated TF and CF function in hematopoiesis comes from the observation that both somatic and germline mutations in TF and CF lead to altered hematopoiesis and the development of leukemia [13]. Mutations and chromosomal rearrangements of TF and CF are highly recurrent, almost uniform, features in acute myeloid leukemia (AML) [13−15]. However, the exact identity and nature of specific TF-CF interactions required to generate the gene regulatory complex (GRC) molecular machinery necessary for transcription, the molecular mechanisms governing these GRCs in both normal HSPC function and differentiation, and their subversion in malignancy are still unresolved.

A roadmap of TF activity exists within haematopoiesis due to their sequence-based tractability via cognate DNA-binding domains, the presence and documented phenotype of germline and somatic TF mutations, available ChIP-Seq maps and the study of individual TFs via genome-engineered murine knockout models. To further address the fundamental role of CF and wider GRCs during normal hematopoiesis, we used clustered regularly interspaced short palindromic repeats (CRISPR)-mediated functional genomic knockout of multiple chromatin factors in vivo and ex vivo. These results demonstrated roles for multiple CFs in specific lineage priming and differentiation, as well as functional diversity within complexes [16]. Disruption of specific CFs led variably to differentiation block and an expansion of progenitor cells by regulation of local chromatin accessibility and the binding of key lineage-determining TFs. The screen also demonstrated specific interactions between CFs, resulting in the recruitment and remodeling of chromatin, likely via both direct protein-protein interactions and posttranslational histone modification. Moreover, our data demonstrated differences between CF and TF associations in the transition between normal and malignant hematopoiesis, suggesting that these complex regulatory interactions can be hijacked in leukemia to facilitate differentiation block and likely other leukemia phenotypic characteristics [16]. Differential usage and differing compositions of GRCs also raise the attractive possibility of targeting leukemia-specific GRCs for therapeutic gain. This review will focus on the members of GRCs, their role in normal hematopoiesis, and how they may be subverted in leukemia.

## Chromatin and DNA Readers, Writers, and Erasers

**Figure 1:**
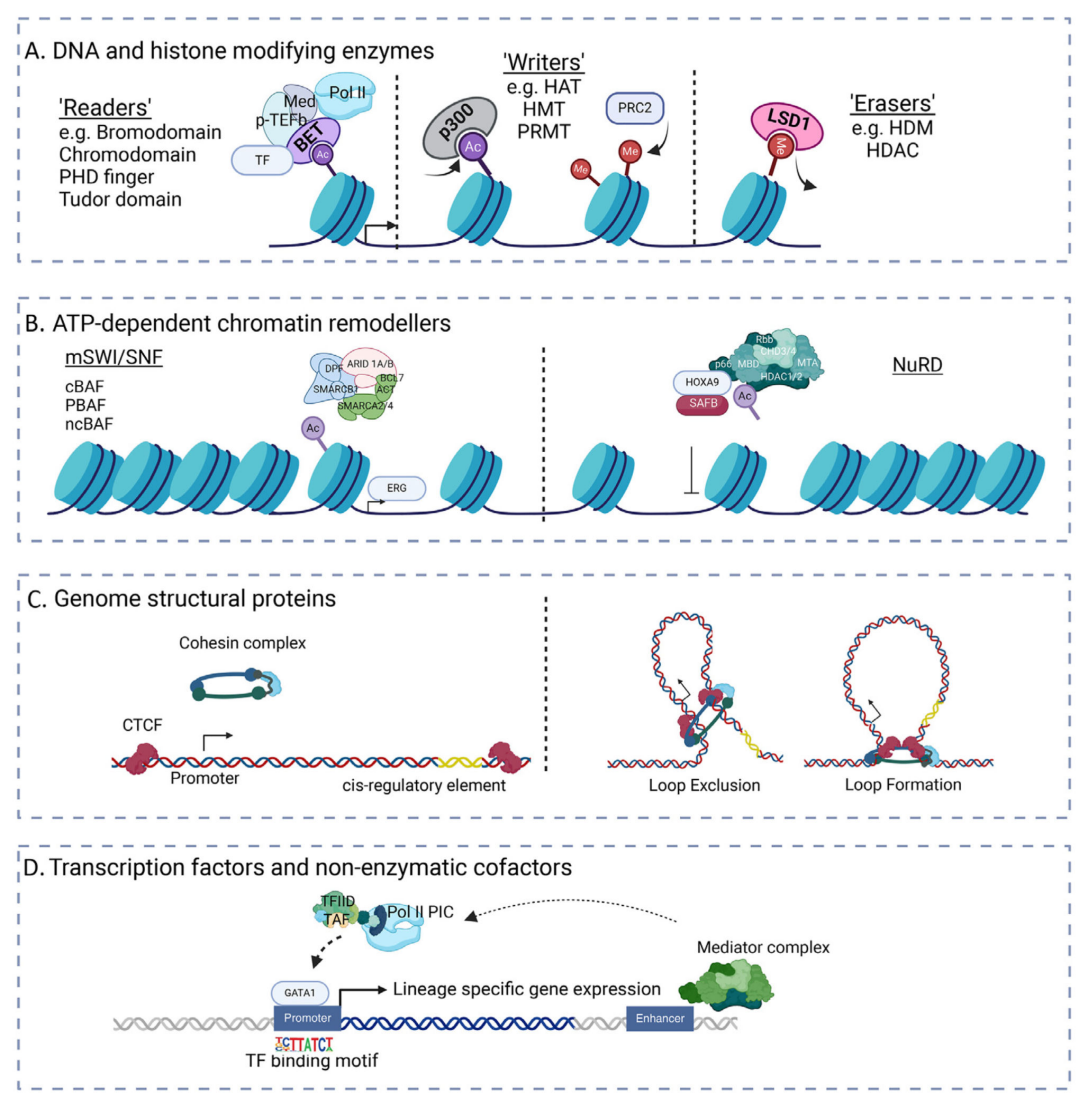
Mechanisms of transcriptional regulation. **(A)** Enzymatic modification of DNA and histones by epigenetic “readers,” “writers,” and “erasers”. **(B)** Schematic of the mechanisms of chromatin accessibility and nucleosome remodeling by mammalian SWI/SNF and NuRD complexes. **(C)** Schematic illustrating the mechanism of loop extrusion to form chromatin loops allowing the communication of promoters and enhancers by the cohesin complex and CTCF. **(D)** Schematic of the formation of a transcription complex with transcription factor GATA1 binding a sequence-specific motif at the promoter and interacting with TAF, TFIID, and the basal transcription machinery. A Mediator complex at the enhancer can interact with the transcription complex to regulate transcription. *cBAF=*BRG1/BRM-associated factor canonical complex; *CTCF*=CCCTC-binding factor; *GATA1*=GATA binding protein 1; *HAT=* histone acetyltransferase; *HDAC=*Histone deacetylase; *HDM=*Histone demethylase; *HMT=* Histone methyltransferase; *ncBAF=*Noncanonical BRG1/BRM-associated factor complex; *NuRD=*Nucleosome remodeling and deacetylase complex; *PBAF=*Polybromoassociated factor complex; *PIC=*Preinitiation complex; *PRMT=*Protein arginine methyltransferase; *SWI/SNF*=Switch/sucrose nonfermentable; *TAF=*TBP-associated factor; *TF=*Transcription factor; *TFIID=*Transcription factor IID. Created with Biorender.com.

Chromatin consists of interacting DNA and proteins, whose basic unit is the nucleosome. This structure consists of ~147 bp of DNA wrapped around an octamer of histone proteins (each consisting of a tetramer of H3-H4 and two H2A-H2B dimers). Many CFs alter epigenetic function by modifying either the proteins (histones) or the DNA of chromatin; they can be classified as those that lay down modifications (DNA or chromatin writers) or those that remove these marks (DNA or chromatin erasers). A third class of protein reads unmodified or specific modifications of DNA and histones, so-called DNA or chromatin readers (Figure 1A).

### DNA Methylation

Methylation of DNA at the 5’ cytosine is classically associated with CpG islands at gene promoters and gene repression. DNA methyltransferases (DNMT) mediate de novo and maintenance cytosine methylation, resulting in gene silencing and transcriptional repression during lineage commitment. Similarly, many genes undergo selective DNA demethylation by 5-methylcytosine dioxygenase ten-eleven translocation (TET) proteins, resulting in transcriptional upregulation [17]. Recurrent mutations in the de novo methyltransferase *DNMT3A* and *TET2* are seen in ~25% and 7%−10% of patients with AML, respectively, and represent the most common mutations associated with clonal hematopoiesis [18]. The mechanism by which these mutations lead to leukemia is not fully understood, but altered methylation signatures are characteristic of AML and can be used to define biological subtypes and predict clinical outcomes [19]. Mechanistic interactions between DNMT enzymes and other chromatin regulators are likely critical to normal function, such as interactions with the polycomb repressive complex 2 (PRC2) complex. Enhancer of zeste homolog 2 (EZH2) has been shown to directly recruit DNMTs to PRC2-repressed genes and appears to be an important driver of DNA methylation [20−23]. Recurrent mutations in both EZH2 and DNMTs in leukemia suggest an underlying mutational synergy in epigenetic regulators that contributes to leukemogenic transcription.

### Histone Modifications

Modifications of histones usually occur within their unstructured tails. There are multiple modifications, but the best understood are acetylation (ac), methylation (me), ubiquitination (Ub), and phosphorylation (P). These alter local chromatin accessibility and the recruitment of other regulatory “reader” proteins to alter DNA-templated processes, particularly gene expression. The polycomb group (PcG) protein complex PRC2 is a highly conserved histone H3 methyltransferase that regulates the expression of developmental genes. The catalytic subunit of PRC2, EZH2, catalyzes trimethylation of histone H3 lysine 27 (H3K27) (H3K27me3) at proximal promoter and distal enhancer cis-regulatory elements, resulting in transcriptional silencing [24]. Inactivating mutations in *EZH2* are observed in myeloid malignancies, and associated upregulation of transcriptional programs conferring chemotherapy resistance points to its role as a tumor suppressor [25−27]. In mice, the loss of *Ezh2* during leukemia induction leads to the resolution of a small number of ‘bivalent’ promoters, which harbor both repressive H3K27 trimethyl and activating H3K4 trimethyl marks [28]. The result is the upregulation of fetal transcriptional programs conferring oncogenicity [28]. However, overexpression of *Ezh2* during established AML also results in AML maintenance, highlighting a context-specific role of chromatin-modifying proteins in leukemogenesis [28].

**Figure 2:**
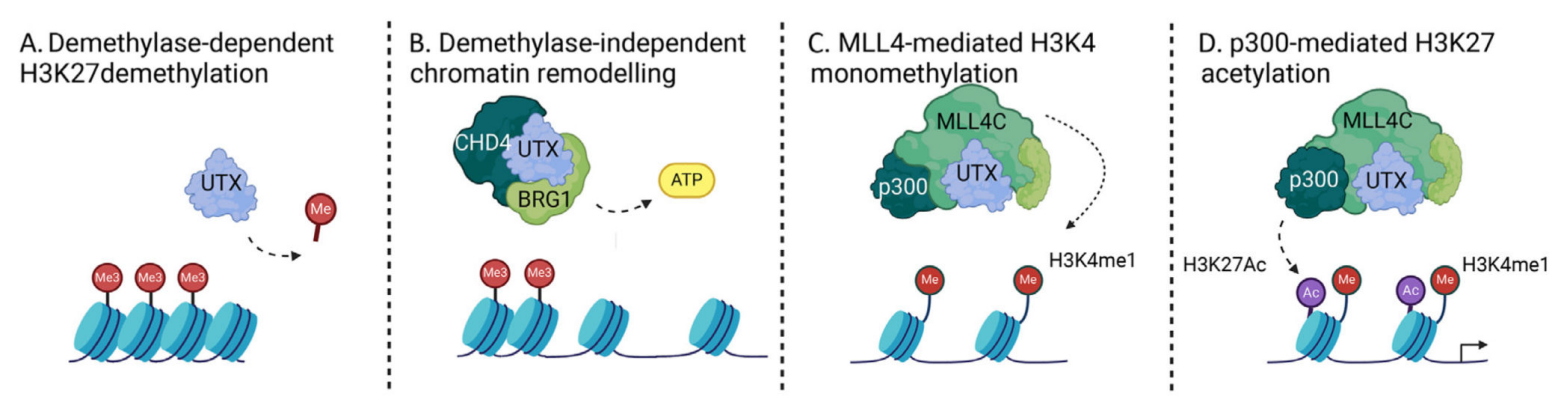
Sequential enhancer licensing by the UTX-MLL4-p300 gene regulatory complex. Schematic illustrating the role of a UTX-MLL4-p300 gene regulatory complex in transcriptional regulation through enhancer licensing. **(A)** Demethylation of H3K27 trimethylation by UTX. **(B)** Demethylase-independent chromatin remodeling by UTX in combination with ATP-dependent chromatin remodeling factors, such as BRG1 and CHD4-containing complexes. **(C)** Recruitment of MLL4 complexes by UTX and H3K4 monomethylation. **(D)** H3K27acetylation by p300 in complex with UTX and MLL4. *CHD*=Chromodomain helicase DNA-binding 4 protein; *BRG1*=Brahma-related gene 1; *H3K27*=Histone H3 lysine 27; *MLL4C=*Mixed lineage leukemia protein 1 through 4 complex; *UTX*=Ubiquitously transcribed tetratricopeptide repeat on chromosome X. Created with Biorender.com.

The Trithorax group (TrxG) proteins have opposing roles to the PcG proteins in gene regulation and include the COMPASS family of histone methyltransferases (SET domain containing 1A [SET1A], SET1B, and mixed lineage leukemia [MLL] proteins 1 through 4 [MLL1-4]) involved in histone H3 lysine 4 [H3K4] methylation and transcriptional activation. MLL complexes deposit H3K4me3 at promoters of developmental genes and bivalent promoters [29,30]. MLL2 is found in a complex with H3K27me3-specific demethylase UTX, indicating an elegant mechanism by which the removal of a repressive mark is accompanied by the deposition of an activating mark to mediate transcriptional activation at *Hox* promoters [31,32]. Epigenetic crosstalk also occurs between MLL4, UTX, and histone acetyltransferase (HAT) p300/CBP at enhancers, resulting in H3K4 monomethylation and H3K27 acetylation in a UTX-dependent manner, licensing active enhancers (Figure 2) [33,34]. Multiple COMPASS H3K4 methyltransferase complexes are required for hematopoiesis and functional screens have demonstrated that perturbation of these results in marked functional diversity, suggesting nonredundant roles of individual complex members. More than 50 different chromosomal translocations involving the fusion of the N-terminus of MLL have been identified, resulting in the leukemic transformation of HPSCs to AML, acute lymphoblastic leukemia (ALL), or biphenotypic leukemia [35]. These fusion proteins form large multimeric complexes, often containing the super-elongation complex or disruptor of telomeric silencing 1-like (DOT1L) complex members. These abnormal complexes facilitate aberrant transcriptional elongation and/or inappropriately recruit other TFs and chromatin-regulatory proteins to MLL-target genes, including the *HOXA* cluster and *MEIS1*, to facilitate leukemic transcription [36−38]. The fusion complex is localized to its target genes in a Menin-lens epithelium-derived growth factor (LEDGF)-dependent manner [39]. Menin is a multifunctional scaffold protein that serves as a scaffold between TFs and chromatin modifiers at genes related to hematopoietic proliferation and differentiation, such as *HOX* and *MEIS1*, providing a mechanistic explanation for the efficacy of Menin inhibitors in *MLL*-rearranged and *NPM1*-mutated leukemia. In non-*MLL*-rearranged leukemia, such as *Npm1c/Flt3-ITD*, we have also demonstrated a role for leukemia-specific interactions of COMPASS complexes with TFs resulting in altered accessibility at *Stat5a* and *Runx2* loci and consequent transcriptional dysregulation [16].

**Figure 3:**
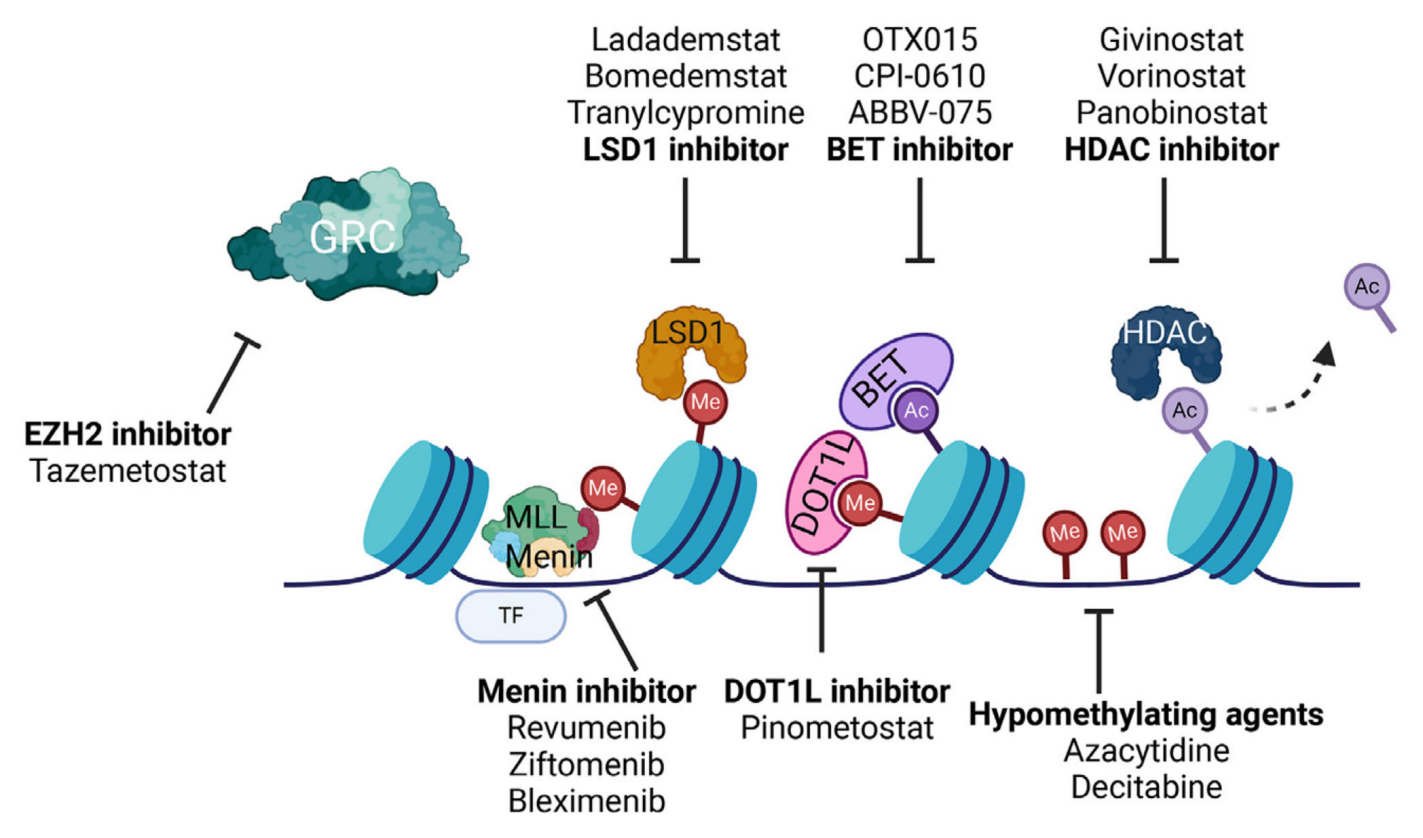
Illustration of mechanisms of targeting GRCs at chromatin. Inhibitors currently under clinical evaluation and their intended chromatin targets are shown. *GRC*= Gene regulatory complex. Created with Biorender.com.

Several chromatin-modifying proteins can modify other histone residues, leading to a diverse array of functional consequences at the chromatin level. These include transcriptional regulation, protein recruitment, and 3D architectural change. Beyond the scope of this review, histone acetylation is dynamically controlled by lysine acetyl-transferases (HATs, e.g., CREB-binding protein [CBP]/E1A binding protein p300 [p300]) and histone deacetylases (HDACs) and is aberrant in AML [40−43]. Though recurrent mutations in HDACs have not thus far been identified in hematologic malignancies, recurrent mutations in CBP/p300 are observed in AML and lymphoid neoplasms [44−46]. Similarly, chromosomal translocations involving lysine acetyltransferases are present in myeloid malignancies [47,48]. Inhibitors of both HATs and HDACs as part of combination therapies represent a promising strategy for specific subtypes of AML (Figure 3) [49,50].

### Epigenetic Readers

Bromodomain and extraterminal (BET) proteins are chromatin regulators that act as epigenetic “readers,” recognizing acetylated lysine residues and performing a critical role in transcriptional regulation (Figure 1A) [51]. Their highly conserved tandem bromodomains are required for the recognition of acetylated histone residues and/or acetylated nonhistone proteins, and the extra-terminal domain may also recruit TFs/chromatin-modifying proteins. Analysis of the BET interactome using proteomic methods has demonstrated interactions with multiple core members of the transcriptional machinery, including the chromatin cofactor Mediator and super-elongation complex [52,53]. Bromodomain-containing protein 4 (BRD4) is a well-recognized transcriptional activator that interacts with the positive transcription elongation factor b (p-TEFb) complex, resulting in the phosphorylation and activation of RNA Pol II, permitting transcriptional elongation [54]. It may also act as a transcriptional repressor through interaction with the PRC2 complex, as well as switch/sucrose nonfermentable (SWI/SNF) and chromodomain helicase DNA-binding 2 (CHD2) proteins [55,56]. BRD4 is a pivotal transcriptional regulator of super-enhancer-associated genes linked to tumor progression, such as *c-MYC*, acting as a scaffold for the recruitment of GRCs that modify chromatin accessibility for TFs. Interruption of this BRD4-dependent transcriptional network is seen after treatment with BET inhibitors, including several critical regulators of myelopoiesis and leukemia, such as *c-MYC, BCL2*, and *IRF8* [57,58]. This effect is observed in *MLL*-rearranged, *NPM1*-mutated, and a diverse array of other AML subtypes (as well as other malignancies), highlighting a role for BET proteins as terminal effectors mediating transcriptional dysregulation in cancer.

## Nonenzymatic Proteins

TFs bind to specific sequences (motifs) within regulatory elements in combination with both enzymatic and nonenzymatic cofactors as well as the basal transcription machinery in a context-dependent manner, forming transcription complexes (Figure 1D). Nonenzymatic cofactors include TATA-binding protein (TBP)-associated factors (TAFs), which are components of the core promoter-recognition complex TF II D (TFIID) and guide the localization of TFs and mediators (Meds). The latter serves as a molecular bridge between enhancer-bound TFs and RNA Pol II, thus facilitating preinitiation complex assembly [59]. For example, interactions between TAF10, Med1, and GATA binding protein 1 (GATA1) mediate the recruitment of TFIID to GATA1-responsive promoters in mouse erythroid cells and are critical for GATA-1-mediated transactivation [60,61].

## Chromatin Remodeling Complexes

### ATP-dependent Chromatin Remodelers

ATP-dependent chromatin remodelers promote or repress transcription by nucleosome modulation and involve four main families: imitation switch (ISWI), CHD, SWI/SNF, and INO80 complexes [62]. This class of proteins is commonly mutated in solid organ malignancies; however, they are not commonly mutated in leukemia and other hematologic malignancies [63]. This phenomenon may relate to the relatively high abundance of mutations in differentially expressed TFs and other CFs, as these interact widely with chromatin remodelers. This relationship is best described for the mammalian SWI/SNF (mSWI/SNF) complexes and NuRD (CHD) complexes (Figure 1B). Mammalian SWI/SNF complexes generally alter chromatin by repositioning nucleosomes or via ejecting octamers or evicting histone dimers. In contrast, CHD complexes remodel nucleosome assembly, assisting with octamer maturation and nucleosome spacing [62]. Mammalian switch/sucrose nonfermenting (SWI/SNF) complexes are composed of the Brahma (BRM)-related gene 1 (BRG1)/BRM-associated factor canonical (cBAF) complexes, the polybromo-associated BAF (PBAF) complexes, and the more recently described noncanonical BAF (ncBAF) complexes, and they predominantly promote chromatin accessibility to TFs [64,65]. Functional genomic studies implicate an important role for cBAF complexes in normal myeloid differentiation. For example, *Smarcd2* knockdown leads to erythroid skewing and accelerated erythropoiesis [16]. Although mutations affecting mSWI/SNF complex members are not recurrent in AML, multiple studies demonstrate a role for complex members in leukemic gene expression. Shi et al. [66] confirmed the role of Brg1/Smarca4 in the maintenance of proliferation and disease progression in mouse models of AML and ALL as well as in patient cell lines, where knockdown of Brg1/Smarca4 and other complex members led to a loss of proliferation, the induction of differentiation, downregulation of oncogenic transcription, particularly *Myc* and *Hoxa9* gene signatures, and prolonged survival in vivo [66]. ChIP-seq analysis demonstrated that the BAF complex was necessary for binding seven TFs, including the ETS-related gene (Erg), purine-rich sequence binding protein 1 (Pu.1), runt-related TF 1 (Runx1), CCAAT-enhancer-binding protein α (Cebpα), and Cebpβ to a regulatory element that formed a DNA loop with the *Myc* promoter. These findings demonstrated cell-type- and leukemia-specific GRC and chromatin conformations leading to oncogenic transcription and leukemia maintenance.

Noncanonical BAF (ncBAF) complex members include the bromodomain-containing protein 9 (Brd9). Knockdown of *Brd9* reveals a strong dependency on this factor in normal hematopoiesis, resulting in an expansion of progenitors, blockade in B-cell development and terminal myeloid maturation [16]. This knockdown is accompanied by loss of accessibility at myeloid maturation loci and motifs for TFs affecting myeloid maturation (Cebp and activator protein 1 [AP-1]) alongside an increase in accessibility for progenitor-associated TF motifs (Gata2) and at critical loci (*Hoxa7, Hoxa9, Hoxa10*, and *Meis1*). *SF3B1* mutations, which are prevalent in myelodysplastic syndrome (MDS) and leukemia, result in missplicing of Brd9 and a subsequent loss of ncBAF at CTCF-associated loci [67]. This process promoted transformation in melanoma and pancreatic cancer lines, further suggesting a potential role in early malignant progression [67]. In established AML cell lines, however, Brd9 has been shown to be required for growth. Inhibition of BRD9 resulted in a reduction in accessibility at *GATA, AP-1, and ETS* motifs, as well as significantly reduced occupancy of BRD9 and BRG1 at AML-specific enhancers, such as *Myc*, resulting in a strong reduction in chromatin accessibility at enhancers and promoters and enhancer RNA transcription [68]. Accessibility for TP53-binding motifs, however, increased upon inhibition. These data demonstrate both activating and repressive roles for ncBAF members in transcriptional regulation, likely to be determined by cell-type-specific levels of expression, posttranslational modifications, and interactions with other proteins/noncoding RNAs (ncRNAs) [69].

The nucleosome remodeling and deacetylase (NuRD) multisubunit complex contains conserved ATP-dependent chromatin remodelers CHD3/CHD4 and the epigenetic erasers HDAC1/HDAC2, coupling chromatin remodeling with enzymatic (HDAC) function. Interactions between nucleosome remodeling and HDACs cause gene repression at specific loci and regulate differentiation during lymphoid and myeloid maturation. Deletion of CHD4 results in a reduction in granulopoiesis and lymphopoiesis and an increase in immature abnormal erythroid precursors simulating differentiation block [16,70]. Lineage-defining TFs, such as B-cell lymphoma/leukemia 11B (BCL11B), B-cell lymphoma 6 (BCL6), and Ikaros zinc finger protein 1 (IKAROS), interact with the NuRD complex to bring about its recruitment to DNA and regulate transcription [71]. The interaction between NuRD and friend of GATA protein 1 (FOG-1) is well studied and demonstrates how recruitment of NuRD to GATA1-bound sites can lead to transcriptional repression instead of activation [72]. The remodeling activity of NuRD is also indispensable for γ-globin silencing [73]. Recent functional screens have demonstrated CHD4 as a dependency for leukemia-specific growth, disease maintenance, and *MYC* overexpression, additionally highlighting the role of this remodeling complex in leukemogenesis [74]. Moreover, we have recently demonstrated that Hoxa9-mediated leukemia maintenance requires the recruitment and activity of the NuRD complex to ensure the repression of critical differentiation genes, including *NOTCH1* and *CEBPδ* [75].

## Genome Structural Proteins

Multiple experiments over the last decades have demonstrated that gene expression is not regulated in a linear fashion but involves coordinated 3D rewiring of DNA topology [76,77]. This topology itself relates to the activity of genome structural proteins, particularly the ring-like Cohesin complex and the loop-scaffold protein CTCF, with both factors recurrently mutated in AML.

### Cohesin Complex

Cohesin and CTCF facilitate interactions between cis-regulatory enhancers and promoters and contribute to higher-order chromatin organization into topologically associated domains (TADs) and larger A/B compartments. The ring-like cohesin complex forms DNA “loops” that bring together distal (enhancer) and proximal (promoter) cis-regulatory elements or exclude their contact, through a process called loop extrusion, to regulate gene expression (Figure 1C). Cohesin members and dynamic looping are required for normal hematopoietic differentiation, as the knockout of complex members results in a differentiation block [16,78]. Recurrent mutations in both cohesin and CTCF chromatin remodeling complexes are seen in myeloid malignancies, including AML (~10%-15%), and in more than one half of cases of acute megakaryoblastic leukemia associated with Down Syndrome (DS-AMKL) [14,79]. Despite their critical role in chromosome segregation during mitosis, cohesin mutations are not associated with aneuploidy, suggesting transcriptional dysregulation as the likely mechanism.

Evidence suggests that cohesin mutations result in haploinsufficiency and loss of function, leading to impaired normal differentiation, increased serial replating ability, and enrichment for HSPC and leukemia stem cell gene expression programs. This phenotype relates to alterations in chromatin accessibility in HSPC, resulting in globally decreased chromatin accessibility at transcriptional regulatory elements for lineage-specific genes, while specifically increasing accessibility and binding of TFs *GATA2, RUNX1*, and *ERG* [80,81]. In addition, cohesin depletion leads to a loss of dynamic alterations in DNA looping and a failure to induce differentiation, for example, of the erythroid program, as well as an expansion of hematopoietic progenitors, immature myeloid cells, and erythroid and granulocyte dysplasia [82]. Taken together, these data illustrate a critical link between cohesin subunits, local chromatin architecture, and gene regulation that contributes to hematopoietic transformation.

Cohesin mutations alone are insufficient to drive AML, and co-operation with additional genetic aberrations is required. Mutations in specific subunits are mutually exclusive and associated with different comutation patterns, with *STAG2* being associated with mutations in poor-risk *SRSF2, ASXL1, BCOR, IDH1/2, CEBPA*, and *RUNX1* genes, whereas *RAD-21* mutated AMLs predominantly associate with mutations in *NPM1* and *FLT3* [83,84]. These differential patterns of co-mutation point to independent mechanisms for leukemogenesis and give rise to differences in patient outcomes. Interactions with other chromatin modifiers/regulators are likely required; for example, sustained expression of *HOXA7/A9* occurs in the context of cohesin mutation due to reduced recruitment of PRC2 to these genes [85].

### CTCF

CTCF is a highly conserved, multifunctional zinc finger DNA-binding protein that binds to unmethylated DNA, predominantly at the core sequence CCCTC, where it establishes TAD-boundaries that regulate enhancer/promoter interaction for specific gene expression programs. It also provides an insulator function by constraining some enhancer-promoter interactions and by regulating the spreading of DNA methylation and formation of heterochromatin through interactions with poly(ADP-ribose) polymerase 1 (PARP1) and DNMT1 [86,87]. CTCF mutations, which are recurrent in 2% of AML and 12% of DS-AMKL, have effects on chromatin architecture and gene expression. Mutations in cohesin, CTCF, and *EZH2* occur with similar allele frequencies in DS-AMKL, suggesting a major role for these mutations in its pathogenesis [79]. Cohesin mutations and CTCF mutations are often mutually exclusive, suggesting some functional redundancy, although both co-occur with EZH2 [79].

## Gene Expression Requires the Coordinated Interaction and Activity of Multiple Members in Grcs

As we have detailed above, gene regulation is a complicated and coordinated process. TFs, chromatin remodeling complexes, and chromatin-modifying enzymes do not act in isolation. Combinatorial binding of complex members at the same loci ensures context-dependent alterations in transcriptional output, allowing for complex regulation of lineage specification and transcriptional programs. We have demonstrated this paradigm and how it is subverted in leukemia development using proteomic and ChIP-seq analysis of the H3K27-demethylase UTX, which is mutated at low frequency (~3%) in AML. These multi-omic analyses revealed physical interactions and chromatin co-occupancy between UTX and MLL4 (KMT2D) COMPASS members, BRG1 (SMARCA4), and CHD4 [88]. Deletion of *Utx* led to changes in chromatin occupancy of BRG1 and CHD4, bidirectional changes in chromatin accessibility, H3K27ac, and H3K4me1. However, no significant increase in H3K27me3 was noted, indicating that these changes are due to catalytic-independent mechanisms; presumably protein recruitment and scaffolding activities of UTX. Moreover, ~20%−30% of these putative enhancer regions interacted with promoters, indicating substantial enhancer remodeling after *Utx* loss. Deletion also facilitated the pioneering function of ETS TFs primed to alter gene expression with AML development and coordinate repression of GATA transcriptional targets, imparting preleukemic properties on mutated HSPCs. Mutational synergy is a hallmark of AML, and evaluation of chromatin-regulating proteins, their combinatorial interactions, and the consequent regulation of transcription provides a mechanism for understanding the epigenetic events that drive leukemogenesis.

## Targeting Chromatin Regulators in AML

Multiple small molecule inhibitors have been developed as monotherapies to target chromatin regulators in AML, with varying degrees of efficacy, such as inhibitors of Menin, DOT1L, HDAC, EZH2, Lysine-specific histone demethylase 1 (LSD1), and BET proteins (Figure 3). Developments in functional genomics and increasingly sophisticated CRISPR screens have been utilized to identify redundant combinations of chromatin regulators to increase susceptibility to therapeutic targeting [89]. Due to space considerations, we will focus on the most promising to date as a paradigm: inhibition of the interaction between Menin and the N-terminus of both wild-type (WT) *MLL1* and *MLL1*-fusion proteins. Menin acts as a scaffold protein for the interaction between the MLL1/KMT2A complex and chromatin-modifying factors that are essential for the leukemogenic transcriptional program in AML. This program involves upregulation of the *HOXA* cluster and *MEIS1*, particularly in *KMT2A*-rearranged, *NPM1*-mutated, *NUP98*-rearranged, and *UBTF*-mutated AML. Initial results have shown significant efficacy for monotherapy with Menin inhibitors in relapsed and refractory patients [90,91]. The first-in-human phase II clinical trial (AUGMENT-101) demonstrated a 63% overall response rate and 23% complete response rate with revumenib in heavily pretreated relapsed and refractory patients [90]. Preliminary results with ziftomenib demonstrate complete and overall response rates of 30% and 40%, respectively [92]. These promising findings have led to the initiation of studies combining revumenib with intensive chemotherapy in fit patients (KOMET-008, NCT 05735184) or hypomethylating agents and venetoclax in unfit patients (BEAT AML, NCT03013998) with encouraging results [93]. However, in general, the remissions are not durable, and the emergence of acquired genetic (*MEN1 missense* mutations) and nongenetic resistance mechanisms poses a significant challenge to the prolonged efficacy of Menin inhibition in clinical practice. High-throughput CRISPR screens have demonstrated possible mechanisms of nongenetic resistance, including loss of PRC1.1 subunits and therefore derepression of noncanonical Menin targets, including *MYC*, and a failure of molecular switching to *UTX-KMT2C/D* transcriptional regulation of tumor-suppressive programs upon Menin inhibition [94,95]. Therefore, combinations of Menin inhibitors with other chromatin regulators such as DOT1L, PRC2 complex, and CDK4/6 inhibitors and with intensive chemotherapy or less toxic Ven/Aza regimens are being tested in clinical trials for the treatment of AML [96]. Furthermore, combinations of Menin inhibitors with targeting of other MLL-related chromatin regulators provide promise for overcoming Menin resistance [96].

## Conclusion

Further work to dissect the identity and composition of GRCs that form transcriptional networks in AML is critical for the development of more effective targeted and combination therapies. We know that nongenetic forms of resistance to therapies such as Menin and BET inhibition in AML utilize mechanisms including enhancer switching and transcriptional plasticity to maintain the expression of oncogenes [97]. Targeting the mechanisms of chromatin and enhancer remodeling using rational combinations of epigenetic therapies and epigenetic therapies with standard chemotherapy may help to overcome treatment resistance in AML. Menin inhibition provides a paradigm for the novel mechanism of targeting protein-protein interactions, leading to the partial reversal of transcriptional dysregulation, a cardinal feature of AML.

## Highlights

- Hematopoiesis is governed by the co-ordinated action of transcription factors in complex chromatin factors, resulting in tightly regulated activation and repression of gene expression programs in a spatial and temporal-specific manner.
- Mutations in chromatin and transcription factors and genome structural proteins that regulate lineage commitment are recurrent in acute myeloid leukemia, leading to oncogenic subversion of these transcriptional programs.
- Classical single-gene and high-throughput functional genomic screens have identified individual subunits of these gene regulatory complexes that are vulnerable to therapeutic exploitation.
